# Phosphorylation of the translation initiation factor eIF2*α* at serine 51 determines the cell fate decisions of Akt in response to oxidative stress

**DOI:** 10.1038/cddis.2014.554

**Published:** 2015-01-15

**Authors:** K Rajesh, J Krishnamoorthy, U Kazimierczak, C Tenkerian, A I Papadakis, S Wang, S Huang, A E Koromilas

**Affiliations:** 1 Lady Davis Institute for Medical Research, McGill University, Sir Mortimer B. Davis-Jewish General Hospital, Montreal, Quebec, Canada H3T 1E2; 2 Department of Cancer Immunology, Chair of Medical Biotechnology, Poznan University of Medical Sciences, Poznan, Poland; 3 Division of Experimental Medicine, Department of Medicine, Faculty of Medicine, McGill University, Montreal, Quebec, Canada H3A 1A3; 4 Department of Biochemistry, Faculty of Medicine, McGill University, Montreal, Quebec, Canada H3G 1Y6; 5 Department of Oncology, Faculty of Medicine, McGill University, Montreal, Quebec, Canada H2W 1S6

## Abstract

Phosphorylation of the *α* subunit of the translation initiation factor eIF2 at serine 51 (eIF2*α*P) is a master regulator of cell adaptation to various forms of stress with implications in antitumor treatments with chemotherapeutic drugs. Herein, we demonstrate that genetic loss of the eIF2*α* kinases PERK and GCN2 or impaired eIF2*α*P by genetic means renders immortalized mouse fibroblasts as well as human tumor cells increasingly susceptible to death by oxidative stress. We also show that eIF2*α*P facilitates Akt activation in cells subjected to oxidative insults. However, whereas Akt activation has a pro-survival role in eIF2*α*P-proficient cells, the lesser amount of activated Akt in eIF2*α*P-deficient cells promotes death. At the molecular level, we demonstrate that eIF2*α*P acts through an ATF4-independent mechanism to control Akt activity via the regulation of mTORC1. Specifically, eIF2*α*P downregulates mTORC1 activity, which in turn relieves the feedback inhibition of PI3K resulting in the upregulation of the mTORC2-Akt arm. Inhibition of mTORC1 by rapamycin restores Akt activity in eIF2*α*P-deficient cells but renders them highly susceptible to Akt-mediated death by oxidative stress. Our data demonstrate that eIF2*α*P acts as a molecular switch that dictates either cell survival or death by activated Akt in response to oxidative stress. Hence, we propose that inactivation of eIF2*α*P may be a suitable approach to unleash the killing power of Akt in tumor cells treated with pro-oxidant drugs.

Oxidative stress is a common form of stress caused by the generation of reactive oxygen species (ROS), which are by-products of oxidative protein folding, mitochondrial respiration and detoxification.^1^ Oxidative stress results in the induction of several intracellular pathways owing to the activation of receptor tyrosine kinases and/or inactivation of phosphatases in order to facilitate either cell survival or death.^1^ A key pathway induced by oxidative stress is the phosphatidylinositol 3-kinase (PI3K)-Akt/PKB pathway owing to either the activation of the epidermal growth factor receptor (EGFR) or inactivation of the phosphatase and tensin homolog deleted in chromosome 10 (PTEN).^2^ Akt activity is induced by phosphorylation at threonine (T) 308 and serine (S) 473 mediated by the PI3K-dependent kinase 1 (PDK1) and the mammalian target of rapamycin complex 2 (mTORC2) kinase, respectively.^3^ Many studies have shown that activated Akt assumes a pro-survival role in cells exposed to oxidative insults.^4, 5, 6, 7, 8, 9, 10, 11, 12^ More recent studies, however, revealed an unusual function of Akt, which is its ability to increase the synthesis of intracellular ROS and inhibit proliferation through the induction of either premature senescence or cell death.^13, 14, 15, 16, 17^

An immediate response of cells exposed to various forms of stress is the general inhibition of protein synthesis, which is mainly caused by the increased phosphorylation of the *α* subunit of the translation initiation factor eIF2*α* at S51 (herein referred to as eIF2*α*P).^18^ Induction of eIF2*α*P serves as an important ‘checkpoint', under which general protein synthesis and cell proliferation are blocked, thus allowing cells to either recuperate from stress or be eliminated if the damage is beyond repair.^19^ eIF2*α*P is mediated by a family of kinases consisting of the heme-regulated inhibitor (HRI), which is activated by heme deficiency to inhibit globin synthesis in erythroid cells; PKR, an interferon (IFN)-inducible protein with pro-inflammatory and antiviral properties, which is activated by binding to double-stranded (ds) RNA; the endoplasmic reticulum (ER)-resident protein kinase PERK/PEK, which is activated by the accumulation of misfolded proteins in the ER; and the general control non-derepressible-2 (GCN2), which is activated by accumulation of uncharged tRNAs caused by amino-acid starvation or nutrient deprivation.^19, 20^ Despite the general shutdown of protein synthesis, certain mRNAs similar to those encoding the activating transcription factor 4 (ATF4) and ATF5 in mammals and GCN4 in yeast are efficiently translated under conditions of increased eIF2*α*P through a mechanism that involves delayed translation re-initiation.^21, 22^ Increased translation of ATF4 and ATF5 or GCN4 is required to increase the expression of genes that facilitate the adaptation of cells to stress.^19^

eIF2*α*P has an important role in the regulation of redox homeostasis and adaptation to oxidative stress in different species including humans, mice, worms and yeast.^23, 24, 25^ In metazoans, oxidative stress is closely linked to ER stress, given that accumulation of misfolded proteins in the ER leads to generation of ROS, which in turn can elicit the unfolded protein response (UPR) as a means to protect cells from stress.^1^ UPR activates the PERK-eIF2*α*P arm, which via the translational upregulation of ATF4 mediates the transcriptional induction of genes encoding antioxidant proteins.^23^ PERK can also exhibit an antioxidant effect independent of eIF2*α*P through the phosphorylation and activation of transcription NF-E2-related factor 2 (Nrf2).^26^ The antioxidant function of eIF2*α*P also involves the attenuation of general protein synthesis, which decreases client protein load and prevents illegitimate disulfide bond formation in the ER leading to a sufficient amount of reducing equivalents to alleviate cells from oxidative stress.^23^ In addition, attenuation of protein synthesis by increased eIF2*α*P prevents cells from ATP depletion and stimulation of mitochondrial oxidative phosphorylation and ROS production.^27^ Protein synthesis and ROS production are two processes that are tightly interdependent in stressed cells. That is, ATF4 contributes to restoration of protein synthesis following its inhibition by increased eIF2*α*P in response to ER stress.^28, 29^ However, if restoration of protein synthesis occurs before the recovery of protein-folding capacity of the ER, increased ROS production by protein misfolding utilizes ATF4 to orchestrate a pro-apoptotic program that selectively eliminates stressed cells.^28^

We recently demonstrated that inactivation of the PERK-eIF2*α*P arm in mouse as well as human primary fibroblasts is associated with increased ROS synthesis and induction of premature senescence.^30^ We noticed that unlike primary cells, which were intolerant to increased ROS levels, immortalized as well as tumor cells-deficient in eIF2*α*P were adapted to increased ROS production.^30^ This is consistent with other studies showing that tumor cells are tolerant to ROS up to a certain level, above which, they become sensitive to the antiproliferative effects of excessive oxidative stress.^31, 32^ Cells engage several mechanisms to become tolerant to ROS some of which depend on Akt.^11, 13^ A functional connection between Akt and eIF2*α*P has been established by our group showing that Akt activation by ER stress depends on eIF2*α*P.^33, 34^ We further showed that activated Akt in turn acts in a negative regulatory loop to decrease eIF2*α*P through the inactivation of PERK and GCN2.^33, 35^ On the basis of these findings, we were interested to examine the roles of eIF2*α*P and Akt in determining the sensitivity of immortalized and tumor cells to oxidative stress. Herein, we provide strong evidence that the cell fate function of eIF2*α*P is mediated by the regulation of Akt activity. Specifically, we found that eIF2*α*P promotes Akt activation to facilitate cell survival under oxidative stress. Although impaired eIF2*α*P diminishes Akt activation, the remainder of activated Akt in eIF2*α*P-deficient cells facilitates death in response to oxidative stress.

## Results

### eIF2*α*P promotes cell survival in response to oxidative stress

**Figure 1:**
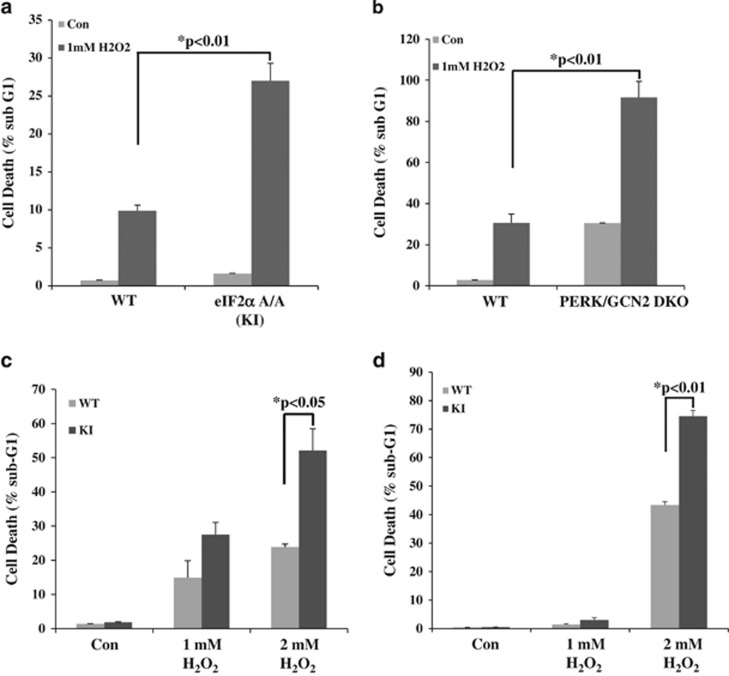
Deletion of PERK and GCN2 or impaired eIF2*α*P promotes cell death by oxidative stress. eIF2*α*P-deficient (KI) MEFs (**a**) or PERK and GCN2 double knock-out (DKO) MEFs together with their isogenic wild-type (WT) counterparts were treated with 1 mM H_2_O_2_ for 6 h. Human fibrosarcoma HT1080 (**c**) or lung adenocarcinoma A549 cells (**d**) that were either proficient (WT) or deficient in eIF2*α*P (KI) were treated with indicated concentrations of H_2_O_2_ for 8 h. Cell death was assessed by the percentage of cells in sub-G_1_ population by propidium iodide staining and FACS analysis. Histograms represent the quantification from three independent experiments performed in triplicates. Error bars represent the S.E.

We observed that immortalized mouse embryonic fibroblasts (MEFs) expressing a serine 51 to alanine (S51A) mutant of eIF2*α*P (herein referred to as knock-in (KI) cells) were highly susceptible to death by hydrogen peroxide (H_2_O_2_) treatment compared with isogenic MEFs expressing wild-type (WT) eIF2*α* (Figure 1a). Previous studies showed that among the eIF2*α* kinases PERK and GCN2 promote survival, whereas PKR induces death in different cell types exposed to oxidative stress.^23, 36, 37, 38, 39^ On the other hand, HRI has had a more specialized role by promoting the survival of primary erythroid precursors subjected to oxidative stress.^40^ We obtained evidence that knockout (KO) of both PERK and GCN2 was required to render MEFs increasingly susceptible to death by H_2_O_2_ treatment compared with WT control cells (Figure 1b). In addition, whereas eIF2*α*P KI and PERK/GNC2 KO MEFs were increasingly susceptible to H_2_O_2_ treatment compared with their isogenic control counterparts, the amount of cell death caused by the loss of both PERK and GCN2 was higher than the amount of death caused by impaired eIF2*α*P (Figures 1a and b). This phenomenon may be due to differences in the genetic background as previously shown by our group for MEFs subjected to stress by glucose deprivation.^41^ It is also possible that PERK and GNC2 promote cell survival under oxidative stress via eIF2*α*P-dependent as well as -independent pathways as shown previously by the ability of PERK to mediate the activation of Nrf2.^26^ These data suggested that the pro-survival effects of increased eIF2*α*P in MEFs exposed to oxidative stress depend on the activation of PERK and GCN2.

**Figure 2:**
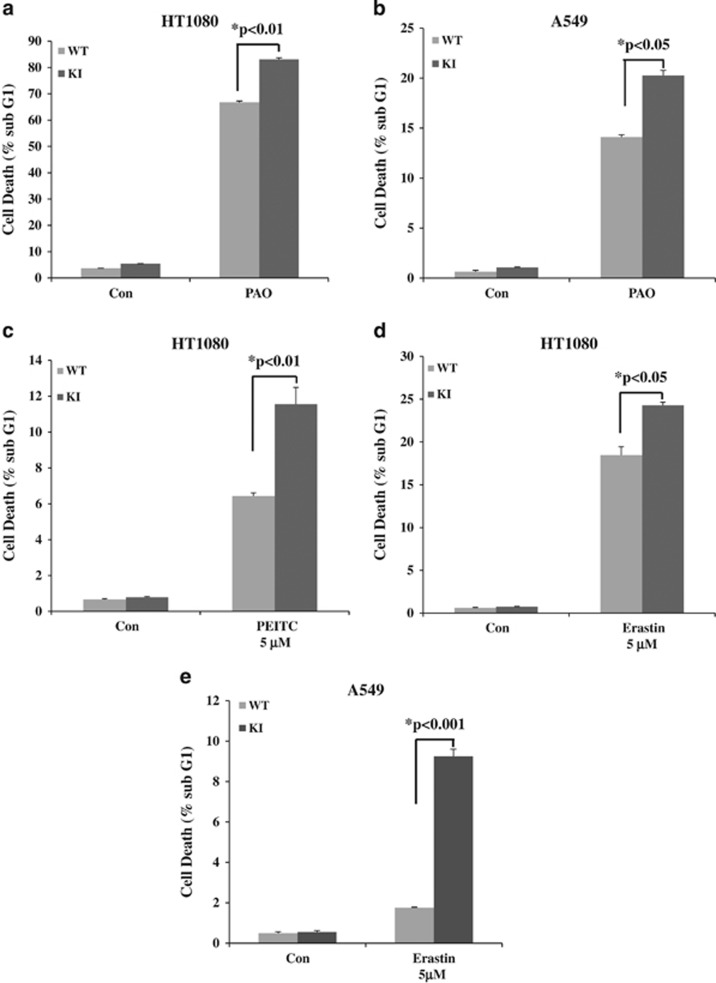
Impaired eIF2*α*P sensitizes human tumor cells to death with pro-oxidant drugs. HT1080 and A549 cells that were either proficient (WT) or deficient in eIF2*α*P (KI) were treated with 2 *μ*M of the pro-oxidant drug PAO (**a** and **b**) for 6 h or with indicated concentrations of PEITC) (**c**) or erastin (**d** and **e**) for 24 h. Histograms represent the percentage of cell death as indicated by the sub-G_1_ population derived from two independent experiments performed in triplicates. Error bars represent the S.E.

Several studies have supported the notion that tumor cells are tolerant to intrinsic oxidative stress caused by increased ROS synthesis but become increasingly sensitive to extrinsic oxidative insults.^32, 42^ To determine the role of eIF2*α*P in the sensitivity of tumor cells to oxidative stress, we employed human fibrosarcoma HT1080 cells and lung adenocarcinoma A549 cells, which were either proficient (WT) or deficient (KI cells) in eIF2*α*P.^30^ Specifically, human tumor cells were made deficient in eIF2*α*P by infection with retroviruses expressing an HA-tagged form of the phosphorylation-defective eIF2*α*S51A followed by infection with lentiviruses targeting the 3′ UTR of endogenous eIF2*α*.^30^ We observed that eIF2*α*P-deficient cells were more sensitive to death by H_2_O_2_ treatment than eIF2*α*P-proficient cells (Figures 1c and d). The increased sensitivity of the eIF2*α*P-deficient tumor cells to oxidative stress was also observed when cells were treated with pro-oxidant drugs such as the cysteine oxidant phenylarsine oxide (PAO)^43, 44^ or *β*-phenylethyl isothiocyanate (PEITC), which disables the glutathione antioxidant system (Figures 2a–c).^13, 45^ eIF2*α*P-deficient HT1080 cells were also increasingly susceptible to death by erastin, a pro-oxidant drug that preferentially kills tumor cells with activating *ras* mutations (Figure 2d).^46^ These data further supported the antioxidant and pro-survival functions of eIF2*α*P in tumor cells subjected to different forms of oxidative insults.

### eIF2*α*P deficiency compromises Akt activation in cells subjected to oxidative stress

**Figure 3:**
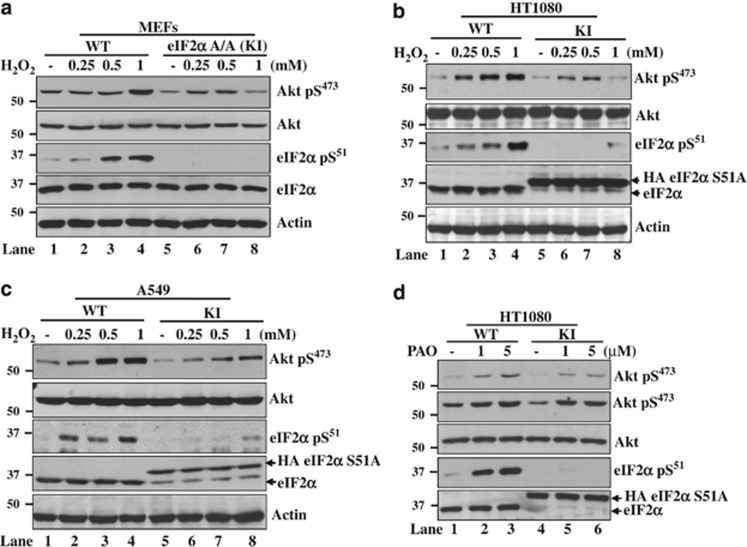
eIF2*α*P promotes Akt activation in response to oxidative stress. eIF2*α*P-proficient as well as -deficient immortalized MEFs (**a**), HT1080 cells (**b** and **d**) or A549 cells (**c**) were exposed to the indicated concentrations of either H_2_O_2_ (**a**–**c**) for 2 h or PAO for 15 min (**d**). Protein extracts (50 *μ*g) were immunoblotted for the indicated proteins. The decreased migration of HA-eIF2*α*S51A in KI cells compared with endogenous eIF2*α* is indicated (**b** and **c**, lanes 5–8; **d**, lanes 4–6)

**Figure 4:**
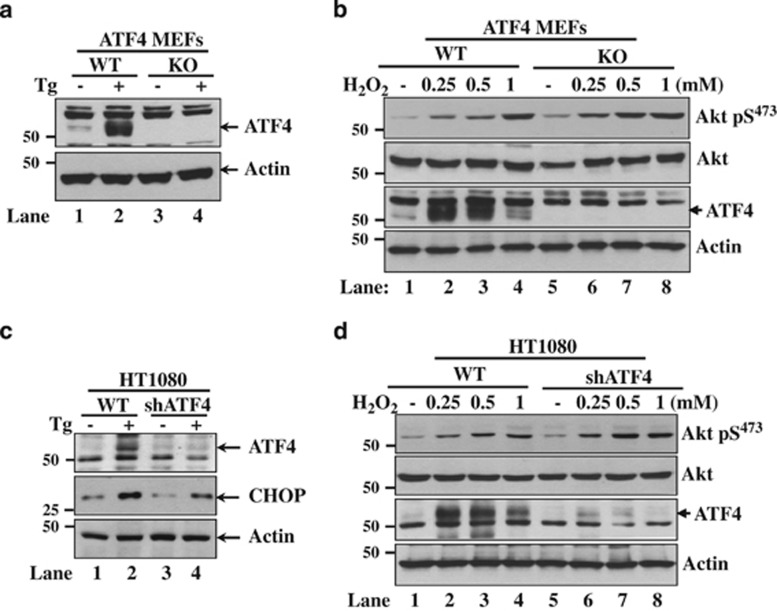
Akt activation by oxidative stress occurs independent of ATF4. MEFs (**a** and **b**) or HT1080 cells, which were either proficient (WT) or deficient in ATF4 by gene KO (**a** and **b**) or shRNA expression (**c** and **d**), were treated with 1 *μ*M thapsigargin (Tg) for 4 h (**a** and **c**) or exposed to indicated amounts of H_2_O_2_ for 2 h. Protein extracts (50 *μ*g) were subjected to immunoblot analysis for the indicated proteins. Decreased levels of C/EBP homologous protein (CHOP) were used as a marker of ATF4 inactivation (**c**)

An important mechanism utilized by cells to respond to oxidative insults involves the activation of Akt, which can promote cell survival or death.^14^ Previous work by our group showed that induction of eIF2*α*P in response to ER stress leads to the activation of the PI3K-Akt pathway as a means to protect cells from stress.^34^ We found that H_2_O_2_ treatment resulted in a higher induction of Akt S473 phosphorylation in eIF2*α*P-proficient than -deficient MEFs (Figure 3a). The ability of eIF2*α*P to facilitate Akt S473 phosphorylation was further verified in eIF2*α*P-proficient and -deficient HT1080 or A549 tumor cells exposed to either H_2_O_2_ or pro-oxidant drug PAO (Figures 3b–d). Because ATF4 is an important mediator of the antioxidant function of eIF2*α*P,^23^ we examined whether ATF4 has a role in Akt regulation in response to oxidative stress. We found that ATF4 inactivation by either gene KO in MEFs or knockdown by the shRNA approach in HT1080 cells did not impair Akt S473 phosphorylation after H_2_O_2_ treatment (Figures 4a–d). These data indicated that Akt activation by eIF2*α*P in cells subjected to oxidative stress occurs via an ATF4-independent pathway.

**Figure 5:**
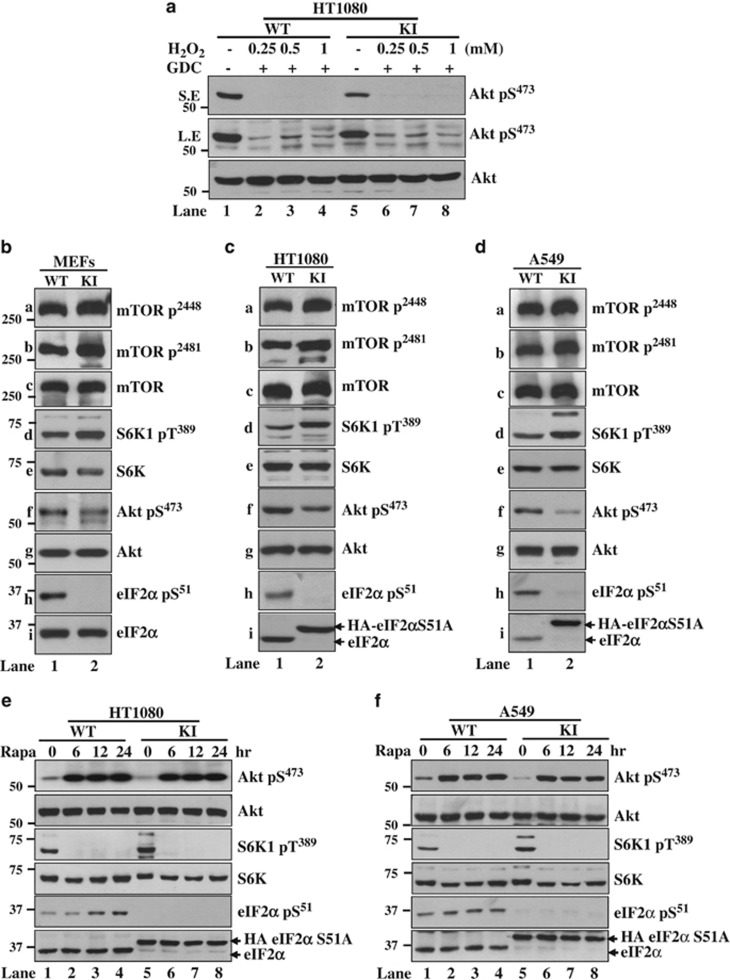
eIF2*α*P promotes Akt activation via mTORC1 inactivation. (**a**) Immunoblot analyses of 50-*μ*g protein extracts from eIF2*α*P-proficient (WT) or deficient (KI) HT1080 cells, which were pretreated with 5 *μ*M GDC-0941 for 30 min followed by treatments with the indicated concentrations of H_2_O_2_ for 2 h. (**b**–**d**) Immunoblot analyses of 50-*μ*g protein extracts from MEFs (**b**), HT1080 cells (**c**) or A549 cells (**d**) that were either proficient (WT) or deficient in eIF2*α*P (KI) in the absence of treatment. (**d** and **e**) Immunoblot analyses of 50-*μ*g protein extracts from HT1080 WT and KI cells (**e**) or A549 WT and KI cells (**f**) before or after treatment with 20 ng/ml rapamycin for the indicated hours

Oxidative stress activates Akt via PI3K-dependent as well as -independent pathways.^47, 48^ To determine the role of PI3K in this process, cells were treated with H_2_O_2_ in the presence of the specific PI3K inhibitor GDC-0941. We observed that GDC-0941 compromised Akt S473 phosphorylation in eIF2*α*P-proficient and -deficient cells indicating that PI3K signaling has a primary role in Akt activation downstream of eIF2*α*P (Figure 5a). A major mechanism of Akt regulation involves mTORC1, which upregulates a negative feedback loop from the ribosomal S6 kinases 1 and 2 (S6K1/2) to insulin receptor substrate (IRS1) resulting in PI3K inhibition.^49, 50, 51^ We observed that eIF2*α*P deficiency in either immortalized MEFs or HT1080 and A549 tumor cells were associated with an increase in mTOR autophosphorylation at S2481 as well as mTOR phosphorylation at S2448 by S6K.^52, 53, 54, 55^ mTORC1 activation was further accompanied by an increase in S6K1 T389 phosphorylation and decreased Akt S473 phosphorylation making evident the presence of the negative regulatory loop downstream of mTORC1 (Figures 5b–d). To further substantiate this observation, cells were treated with the mTORC1 inhibitor rapamycin in order to eliminate the negative feedback regulation of Akt. We found that rapamycin treatment resulted in the induction of Akt S473 phosphorylation in both eIF2*α*P-proficient and -deficient tumor cells at similar levels (Figures 5e and f). Restoration of Akt activity by rapamycin revealed the positive effect of eIF2*α*P on PI3K-Akt signaling via the inactivation of mTORC1.

### eIF2*α*P determines the balance between cell survival and death by activated Akt under oxidative stress

**Figure 6:**
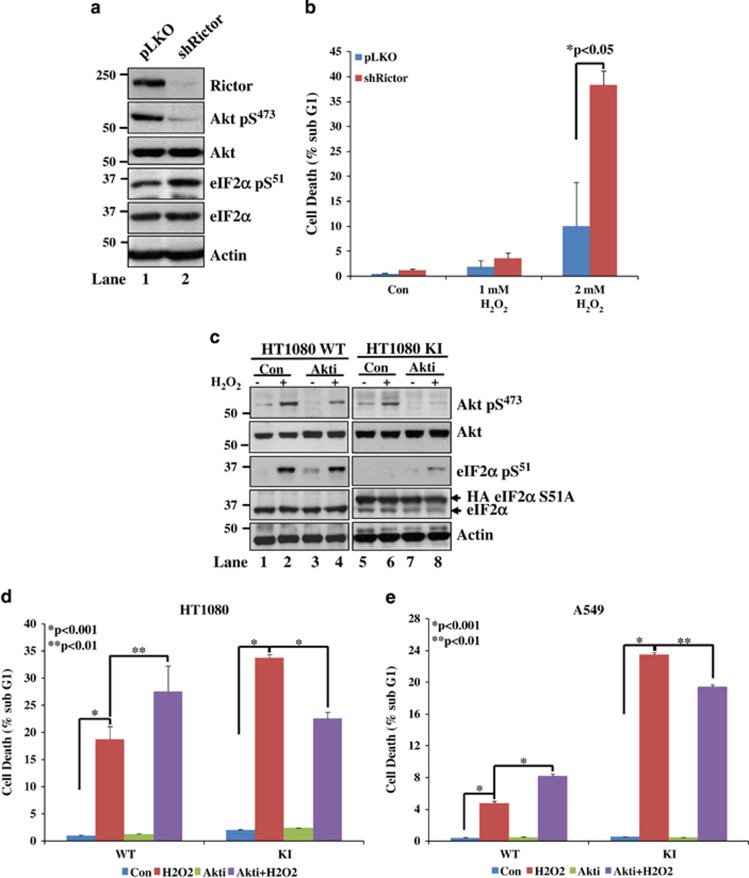
eIF2*α*P determines the cell fate effects of activated Akt in response to oxidative stress. (**a**) HT1080 cells were infected with either insert-less pLKO retroviruses or pLKO retroviruses expressing Rictor shRNA. After puromycin selection, protein extracts (50 *μ*g) were subjected to immunoblot analyses of the indicated proteins. (**b**) HT1080 cells that were either proficient (pLKO) or deficient in Rictor (shRictor) were subjected to treatment with the indicated concentrations of H_2_O_2_ for 6 h. (**c**) HT1080 WT and KI cells were subjected to either single or combined treatments with 1 *μ*M Akt 1,2 (30 min pretreatment) followed by 1 mM H_2_O_2_ for 2 h. Protein extracts (50 *μ*g) were used for immunoblot analyses of the indicated proteins. (**d** and **e**) eIF2*α*P-proficient (WT) or -deficient (KI) HT1080 cells (**d**) or A549 cells (**e**) were subjected to combination treatments with 1 *μ*M Akt 1,2 (30 min pretreatment) and/or 1 mM H_2_O_2_ for 6 h. (**b**, **d** and **e**) Cell death was assessed by the percentage of cells in sub-G_1_ population by propidium iodide staining and FACS analysis. Histograms represent the quantification from three independent experiments performed in triplicates. Error bars represent the S.E.

To better understand the biological role of Akt, we employed HT1080 cells to impair mTORC2 activity by the expression of an shRNA, which was previously shown to cause an efficient downregulation of its Rictor component.^56^ We observed that Rictor impairment led to a substantial reduction of Akt S473 phosphorylation supporting the essential role of mTORC2 in Akt activation by S473 phosphorylation (Figure 6a).^56^ We also noticed that Rictor inactivation caused an increase in eIF2*α*P most likely due to Akt inactivation and inhibition of the feedback inhibitory effects of activated Akt on PERK and GCN2 as shown by our group (Figure 6a).^35^ We further observed that Rictor-deficient HT1080 cells were increasingly susceptible to death by H_2_O_2_ treatment suggesting that Akt activation by S473 phosphorylation has a pro-survival role in eIF2*α*P-proficient cells exposed to oxidative stress (Figure 6b).

We next compared the effects of oxidative stress on eIF2*α*P-proficient and -deficient cells treated with the pharmacological inhibitor Akti-1/2, which impairs the pleckstrin homology domain-dependent function of Akt 1 and 2 isoforms.^57^ We observed that Akti-1/2 decreased Akt S473 phosphorylation in both eIF2*α*P-proficient and -deficient cells after H_2_O_2_ treatment (Figure 6c). The lack of a complete inhibitory effect of the inhibitor on Akt S473 phosphorylation was most likely due to efficient phosphorylation of Akt3 isoform (Figure 6c). We also noticed that Akti-1/2 treatment increased background eIF2*α*P in eIF2*α*P-proficient cells consistent with our interpretation that Akt inhibition relieves the negative regulation of eIF2*α*P (Figure 6c, lane 3).^35^ When the biological effects of Akti-1/2 were tested, we found that Akt inhibition further enhanced the death of eIF2*α*P-proficient HT1080 or A549 tumor cells in response to H_2_O_2_ (Figures 6d and e). This result was in line with a pro-survival role of Akt in response to oxidative stress as also shown by Rictor inactivation (Figure 6b). Interestingly, we noticed that, in contrast to eIF2*α*P-proficient cells, Akt inhibition prevented the death of eIF2*α*P-deficient cells treated with H_2_O_2_ (Figures 6d and e). These findings indicated that the lesser amount of activated Akt in eIF2*α*P-deficient cells promotes death in response to oxidative stress.

**Figure 7:**
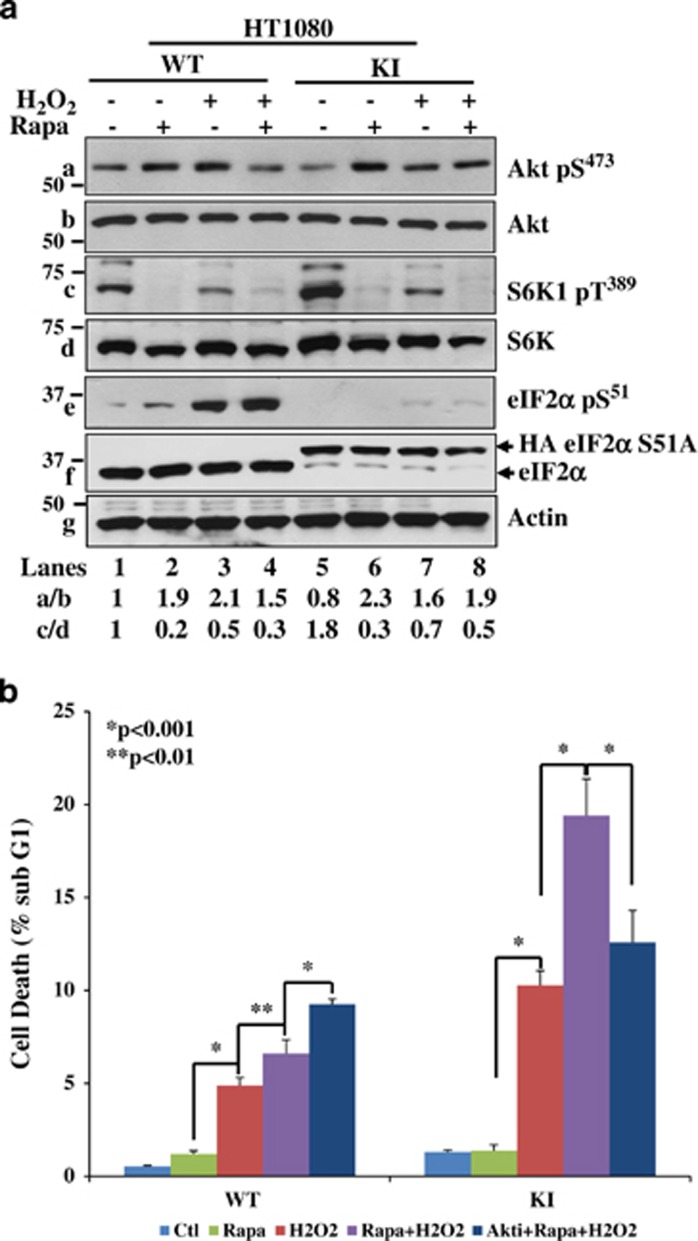
Induction of Akt-mediated death in rapamycin-treated eIF2*α*P-deficient cells subjected to oxidative stress. (**a**) eIF2*α*P-proficient (WT) and -deficient cells (KI) were subjected to either single or combined treatments with 0.5 mM H_2_O_2_ and/or 20 nM rapamycin for up to 2 h. Protein extracts (50 *μ*g) were subjected to immunoblot analyses for the indicated proteins. (**b**) HT1080 WT and KI cells were subjected to either single or combined treatments with 1 *μ*M Akt 1,2 and/or 0.5 mM H_2_O_2_ and/or 20 nM rapamycin for 24 h. Cell death was assessed by the percentage of cells in sub-G_1_ population by propidium iodide staining and FACS analysis. Histograms represent the quantification from three independent experiments performed in triplicates. Error bars represent the S.E.

Previous work showed that mTORC1 inhibition by rapamycin can promote Akt-mediated death in tumor cells subjected to oxidative therapies.^13^ Given that the regulation of Akt activity by eIF2*α*P depends on mTORC1 (Figure 5), we were interested to examine the effects of rapamycin on the sensitivity of eIF2*α*P-proficient and -deficient cells to oxidative stress. We observed that treatment with H_2_O_2_ resulted in the downregulation of mTORC1 in both eIF2*α*P-proficient and -deficient cells as shown by decreased S6K1 T389 phosphorylation (Figure 7a). We also noticed that mTORC1 inhibition by rapamycin restored the differences in Akt S473 phosphorylation in eIF2*α*P-proficient and -deficient cells treated with H_2_O_2_ (Figure 7a). These data indicated that mTORC1 inactivation contributes to eIF2*α*P-mediated Akt activation in cells subjected to oxidative stress (Figure 7a). Concerning the biological effects, rapamycin treatment substantially increased death in both eIF2*α*P-proficient and -deficient cells with the latter cells exhibiting a stronger effect (Figure 7b). These data implied a pro-survival role of mTORC1 in cells subjected to oxidative stress, which was more evident for eIF2*α*P-deficient cells (Figure 7b). Because rapamycin induces Akt activity, we examined the role of Akt by inhibiting its activity in cells subjected to treatments with rapamycin and H_2_O_2_. We found that Akt inhibition by Akti-1,2 treatment further increased the death of eIF2*α*P-proficient cells but substantially decreased the death of eIF2*α*P-deficient cells (Figure 7b). Taken together, these data strongly suggested that (i) mTORC1 conveys a pro-survival function in cells under oxidative stress, which is stronger in eIF2*α*P-deficient than -proficient cells, and (ii) mTORC1 inhibition by rapamycin induces Akt activity to increase the survival of eIF2*α*P-proficient cells and promote the death of eIF2*α*P-deficient cells.

## Discussion

**Figure 8:**
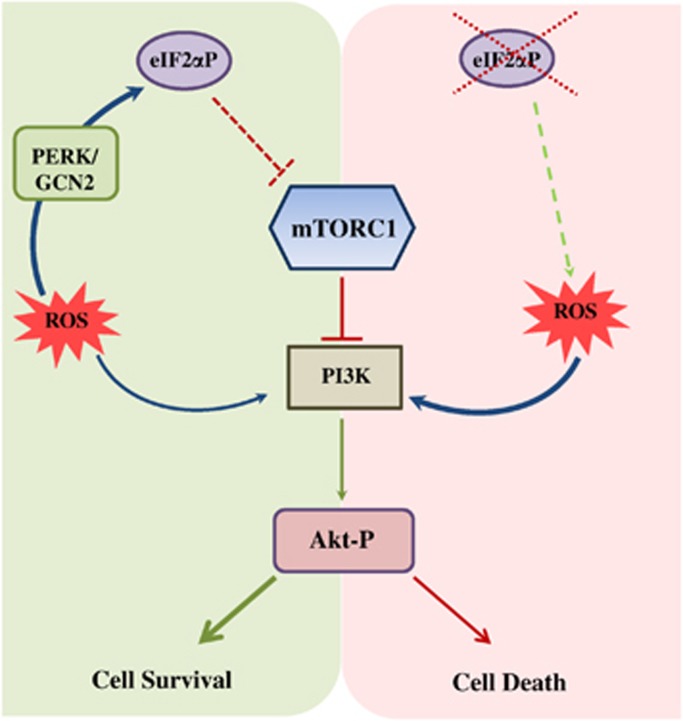
Schematic representation of the functional interactions between eIF2*α*P and Akt in response to oxidative stress. Oxidative stress induces eIF2*α*P and Akt activation through PERK-GCN2 and PI3K, respectively. (Left panel) In cells that are proficient in eIF2*α*P, increased eIF2*α*P decreases mTORC1, which in turn relieves the feedback inhibition of PI3K signaling leading to increased Akt activity and cell survival. (Right panel) In cells that are deficient in eIF2*α*P, mTORC1 activity is upregulated, which in turn diminishes Akt activation through the negative regulation of PI3K signaling. The remainder of activated Akt in these cells promotes cell death in response to oxidative stress. Loss of eIF2*α*P can also lead to increased ROS production,^30^ which may further sensitize cells to extrinsic oxidative stress

Our study uncovers an important role of eIF2*α*P in the regulation of cell fate in response to oxidative stress. Our data support a model in which immortalized as well as tumor cells under oxidative stress induces eIF2*α*P through the activation of PERK and GCN2 (Figure 8, left panel). Increased eIF2*α*P mediates the downregulation of mTORC1 activity, which in turn alleviates the negative regulation of the PI3K-Akt pathway through a previously well-characterized feedback mechanism involving S6K-IRS1.^49, 50, 51^ Decreased mTORC1 activity accounts, at least in part, for the induction of Akt activity in eIF2*α*P-proficient cells under oxidative stress as shown in rapamycin-treated cells (Figure 5). In addition, Akt activation in eIF2*α*P-proficient cells promotes survival inasmuch as its inactivation by genetic or pharmacological means enhances death in response to oxidative stress (Figure 6). On the other hand, impaired eIF2*α*P sensitizes immortalized as well as tumor cells to death after treatment with different forms of pro-oxidant agents (Figure 8, right panel). This is because impaired eIF2*α*P increases mTORC1 activity, which in turn decreases Akt activity owing to upregulation of the feedback loop leading to PI3K inactivation. Impaired eIF2*α*P may further contribute to oxidative stress by upregulating intrinsic ROS synthesis as recently shown by our group.^30^ Despite the downregulation of Akt activity in eIF2*α*P-deficient cells, the remainder of activated Akt promotes cell death in response to oxidative stress (Figure 7). Thus, eIF2*α*P may be an important determinant of the cell fate decisions of activated Akt in cells subjected to oxidative insults.

Other studies have also provided evidence for a functional cross-talk between eIF2*α*P and mTORC1 pathways under different conditions of stress. Specifically, activation of GCN2-ATF4 arm in response to amino-acid deprivation was shown to increase the expression of pyruvate kinase (PKM2), which in turn upregulated mTORC1 activity.^58^ In addition, cells exposed to chronic ER stress were found to induce the PERK-eIF2*α*P-ATF4 arm, which together with mTORC1 was involved in the recovery of mRNA translation under stress.^59^ Furthermore, GCN2-deficient mice displayed enhanced mTORC1 activity and increased sensitivity to oxidative stress caused by asparaginase treatment.^60^ Our data support the notion that the biological function of mTORC1 activation in response to oxidative stress conveys a pro-survival function. This is supported by the induction of death in both eIF2*α*P-proficient and -deficient cells after mTORC1 inhibition by rapamycin (Figure 7b). However, the pro-death effects of rapamycin were affected by Akt and eIF2*α*P inasmuch as Akt inactivation further promoted the killing effects of rapamycin in eIF2*α*P-proficient cells but rescued eIF2*α*P-deficient cells from oxidative death (Figure 7b). Our data are in agreement with previous studies indicating that eIF2*α*P status can determine mTORC1 activation in cells subjected to oxidative stress. Specifically, tethering of mTORC1 to stress granules was shown to prevent mTORC1 hyperactivation in cells subjected to different forms of oxidative stress.^61^ This may be a mechanism by which eIF2*α*P contains mTORC1 activity, given that stress granule formation by oxidative stress depends on eIF2*α*P.^61, 62^ Furthermore, mTORC1 hyperactivation by oxidative stress can have a pro-apoptotic role in cells deficient in tuberous sclerosis complex (TSC).^61, 63^ Given that Akt activity is impaired in TSC-mutant cells, Akt inactivation may be a mechanism by which mTORC1 becomes pro-apoptotic in cells exposed to oxidative stress.

Our data show that induction of eIF2*α*P promotes Akt activation and facilitates cell survival in response to oxidative stress. The data support previous work from our group showing that induction of eIF2*α*P in human tumor cells expressing a conditionally active form of PKR resulted in Akt activation as a means to protect cells from death.^33, 34^ In addition, PERK activation and increased eIF2*α*P by ER stress resulted in the induction of the PI3K-Akt pathway to promote cell survival.^33, 34^ However, it is important to emphasize that, in addition to its ability to activate Akt, eIF2*α*P is also under regulation by activated Akt. Specifically, we recently demonstrated that Akt inactivation by genetic or pharmacological means induces eIF2*α*P via the activation of PERK and GCN2.^33, 35^ This is because PERK and GCN2 are inhibited by Akt-mediated phosphorylation and as such, each eIF2*α* kinase regains full activity under conditions of Akt inactivation.^33, 35^ This process may account for increased eIF2*α*P in tumor cells with impaired Akt S473 phosphorylation as indicated by mTORC2 disruption or pharmacological inhibition of Akt (Figures 6a and c). To date, our work supports a model in which eIF2*α*P and Akt are intertwined in an autoregulatory loop with implications in cell survival under stress.^33^ Specifically, increased eIF2*α*P facilitates the induction of PI3K-Akt signaling, which in turn through the sustained activation of Akt reduces PERK and GCN2 activities as a means to balance eIF2*α*P.^33^ Cells respond to the inhibition of the PI3K-Akt pathway by upregulating eIF2*α*P owing to Akt inactivation and subsequent activation of PERK and GCN2.^33^ In this model, both eIF2*α*P and PI3K-Akt pathways have pro-survival roles with one to substitute for the other under conditions of stress.^33^

Our work may have important implications in antitumor treatments. Specifically, treatment with pro-oxidant drugs is considered an efficient strategy to kill cancer cells that exhibit increased tolerance to ROS.^32, 64, 65^ Because the PI3K-Akt pathway is upregulated in the majority of human cancers, exploiting the pro-death effects of Akt is considered to be an effective strategy in tumor treatment with pro-oxidant drugs.^13^ The pro-death properties of Akt were shown to be mediated by its ability to induce ROS production by increasing oxygen consumption, thereby stimulating oxidative metabolism as well as by repressing the expression of antioxidant genes thorough the inactivation of FoxO transcription factors.^13, 14^ Herein, we provide strong evidence that eIF2*α*P is an important factor in determining the consequence of Akt activation in cells exposed to oxidative stress. Our data show that impaired eIF2*α*P is sufficient to disarm the pro-survival and promote the killing effects of Akt on tumor cells under oxidative stress. Given that inhibitors of the eIF2*α*P pathway have started to emerge,^66, 67, 68^ pharmacological inhibition of eIF2*α*P may hold a promise for the development of strategies that enhance the antitumor effects of pro-oxidant drugs on tumors with hyperactivated Akt.

## Materials and Methods

### Cell culture and treatments

The eIF2*α*P-proficient or -deficient MEFs, HT1080 and A549 tumor cells were generated as described previously.^30^ ATF4 KO MEFs as well as PERK/GCN2 KO MEFs were previously described.^41, 59^ Cells were cultured in Dulbecco modified Eagle medium (Wisent, St-Bruno, QC, Canada) supplemented with 10% fetal bovine serum (FBS; Gibco, Burlington, ON, Canada), antibiotics (100 U/ml of penicillin–streptomycin; Gibco) and 2.5 *μ*g/ml of puromycin (Sigma, Oakville, ON, Canada). The shRNA-mediated KO of ATF4 in HT1080 cells was carried out based on previously reported protocol.^69^ Lentiviral shRNA targeting ATF4 (TRCN0000013573) was obtained from the RNAi Consortium (TRC) arrayed human genome-wide shRNA collection (Sigma). shRNA-mediated KO of Rictor was performed as described previously.^56^ H_2_O_2_ was purchased from Bioshop, Canada; GDC-0941 was obtained from Selleckchem, USA; thapsigargin, rapamycin, Akt1,2 inhibitor, PAO, PEITC and erastin were obtained from Sigma.

### Flow cytometry analysis

Cells were subjected to propidium iodide staining and FACScan analysis based on a previously described protocol.^35^ FACS was performed with BD FACScalibur and the data were analyzed using the FlowJo software (Tree Star Inc., Ashland, OR, USA).

### Western blot analysis

Protein extraction and immunoblotting were performed as described.^30^ The antibodies used were as follows: rabbit monoclonal against phosphorylated eIF2*α* at S51 (Novus Biologicals, Oakville, ON, Canada), mouse monoclonal against eIF2*α*, rabbit monoclonal against phosphorylated Akt at S473, rabbit polyclonal against Akt, rabbit monoclonal against phosphorylated mTOR at S2448 and mTOR, S6K phosphorylated at T389 and S6K, rabbit polyclonal against phosphorylated mTOR at S2481, rabbit polyclonal against CHOP and Rictor were from Cell Signaling Technology (Beverly, MA, USA), rabbit polyclonal against ATF4 (Proteintech, Chicago, IL, USA) and mouse monoclonal antibody against actin (Clone C4, ICN Biomedicals Inc., Irvine, CA, USA). All antibodies were used at a final concentration of 0.1–1 *μ*g/ml. Following incubation with the indicated primary antibodies and washes, membranes were probed with anti-mouse or anti-rabbit IgG antibodies conjugated to horseradish peroxidise (Mandel Scientific, Guelph, ON, Canada). Proteins were visualized with the enhanced chemiluminescence reagent (Thermo Scientific, Waltham, MA, USA) detection system according to the manufacturer's instructions. Quantification of bands in linear range of exposure was performed by densitometry using the Scion image software (Frederick, MD, USA).

### Statistical analysis

Error bars represent S.D. as indicated and significance in differences between arrays of data tested was determined using two-tailed Student *t*-test (Microsoft Excel).

## Abbreviations

ATF: activating transcription factor
GCN: general control non-derepressible
ds RNA: double-stranded RNA
eIF2*α*P: eIF2*α* phosphorylated at S51
ER: endoplasmic reticulum
EGFR: epidermal growth factor receptor
HRI: heme-regulated inhibitor
H_2_O_2_: hydrogen peroxide
IFN: interferon
mTORC: mammalian target of rapamycin complex
MEFs: mouse embryonic fibroblasts
IRS: insulin receptor substrate
KI: knock-in
PDK1: PI3K-dependent kinase 1
PAO: phenylarsine oxide
PEITC: *β*-phenylethyl isothiocyanate
PTEN: phosphatase and tensin homolog deleted in chromosome 10
PI3K: phosphatidylinositol 3-kinase
S6K: ribosomal S6 kinase
ROS: reactive oxygen species
TSC: tuberous sclerosis complex
WT: wild type
